# A Mammalian Lost World in Southwest Europe during the Late Pliocene

**DOI:** 10.1371/journal.pone.0007127

**Published:** 2009-09-23

**Authors:** Alfonso Arribas, Guiomar Garrido, César Viseras, Jesús M. Soria, Sila Pla, José G. Solano, Miguel Garcés, Elisabet Beamud, José S. Carrión

**Affiliations:** 1 Departamento de Investigación en Recursos Geológicos, Instituto Geológico y Minero de España, Madrid, Spain; 2 Departamento de Estratigrafía y Paleontología, Facultad de Ciencias, Universidad de Granada, Granada, Spain; 3 Departamento de Ciencias de la Tierra y del Medio Ambiente, Facultad de Ciencias, Universidad de Alicante, Alicante, Spain; 4 Departamento de Estratigrafía, Paleontología y Geociencias Marinas, Facultad de Geología, Universidad de Barcelona, Barcelona, Spain; 5 Paleomagnetic Laboratory (UB-CSIC) Institute of Earth Sciences, Jaume Almera, Barcelona, Spain; 6 Departamento de Biología Vegetal, Facultad de Biología, Universidad de Murcia, Murcia, Spain; Paleontological Institute, Russian Federation

## Abstract

**Background:**

Over the last decades, there has been an increasing interest on the chronology, distribution and mammal taxonomy (including hominins) related with the faunal turnovers that took place around the Pliocene-Pleistocene transition [ca. 1.8 mega-annum (Ma)] in Europe. However, these turnovers are not fully understood due to: the precarious nature of the period's fossil record; the “non-coexistence” in this record of many of the species involved; and the enormous geographical area encompassed. This palaeontological information gap can now be in part bridged with data from the Fonelas P-1 site (Granada, Spain), whose faunal composition and late Upper Pliocene date shed light on some of the problems concerning the timing and geography of the dispersals.

**Methodology/Principal Findings:**

This rich fossil site yielded 32 species of mammals, among which autochthonous species of the European Upper Villafranchian coexist with canids (*Canis*), ovibovines (*Praeovibos*) and giraffids (*Mitilanotherium*) from Asia. Typical African species, such as the brown hyena (*Hyaena brunnea*) and the bush pig (*Potamochoerus*) are also present.

**Conclusions/Significance:**

This assemblage is taxonomically and palaeobiogeographically unique, and suggests that fewer dispersal events than was previously thought (possibly only one close to 2.0 Ma) are responsible for the changes seen around 1.9–1.7 Ma ago in the fauna of the two continents.

## Introduction

Lying within the western extreme of the Palaearctic, the Iberian Peninsula is known for palaeoenvironmental sites with evolutionary implications of paramount importance. Over long periods of geological time, this has been a land of transitions and physiographical heterogeneity, including the possible existence of islands in the Straits of Gibraltar (enabling exchanges with the African continent). Conceivably, throughout the Cenozoic, climatically influenced species turnover, invasions, and competitive exclusion combined with species survival produced unique associations of plant and animal species. Here, we report on the chronology and composition of the late Upper Pliocene Fonelas P-1 fossil assemblage. Analogous assemblages have not been documented in Eurasia and no other findings have been recovered in the Quaternary. This truly is a large mammal “Lost World”.

### European Mammalian Distribution at the Pliocene/Pleistocene Boundary

Research on the European faunas and environments of the Plio-Pleistocene received an important boost at the end of the 1980's when the first human fossils were identified in the Caucasian deposits of Dmanisi (Zone MNQ -Mammifères Néogènes et Quaternaires-18; Republic of Georgia) [1]). These, and other discoveries related to more recent times in Spain (Orce, Zone MNQ20 [2]; Atapuerca, Zones MNQ21-MNQ20 [3], [4]) and Italy (Ceprano, Zone MNQ21 [5]; Pirro Nord, Zone MNQ20 [6]) have encouraged the research for new human remains at the onset of the Quaternary and the characterization of the ecosystems in which European humans lived or may have lived [7]–[9].

In western Europe, especially in the Iberian Peninsula, little is known about the fauna of the Neogene-Quaternary boundary (N–Q; 1.8 Ma). Both slightly older (Zone MNQ17: La Puebla de Valverde [10]; Huélago [11]) and slightly younger (Zone MNQ20: Venta Micena [12]) records of large mammals exist but no reliable data for the boundary interval were available until 2001 [13]. In order to fill this gap a common practice was to extrapolate to Spain information from nearby European countries (for example, France: Saint Vallier, Zone MNQ17 [14]; Senèze, Zone MNQ18 [15]; Peyrolles, Zone MNQ19 [16] or Italy: Olivola and Tasso Faunal Units (FU's; Zones MNQ18 and MNQ19 [17])). As a result, errors and uncertainties were also extrapolated, especially where taxonomic interpretation and biostratigraphic inferences are concerned. While analyzing any kind of biochronological information, it is however important to bear in mind that discontinuities in the continental records, paleoenvironmental conditions, and taphonomic biases may influence the stratigraphical position of fossil remains, and therefore, the lowest and highest occurrences will not necessarily reflect the temporal order of actual first or last appearances taxa in time [18]. It is equally important to integrate geologic, geomorphologic and tectonic information within a regional context, both as concerns fossiliferous sedimentary basins and the surroundings reliefs. Yet, it is crucial to provide detailed taxonomic analyses based on anatomical variables and including a nomenclatural treatment. Ideally, too, all of the available information should be continuously updated and stored in accessible databases. Otherwise, the inferred biochronological pictures will be strongly wrinkled [19].

The N–Q boundary in Atlantic regions of Europe remains essentially unknown in terms of terrestrial ecosystems, the Late Upper Pliocene faunal diversity, and large mammal assemblages. Which animal species inhabited these territories? Where did some of them come from? Did they coexist with hominins? These questions remain unanswered, even when data is available for the natural events that took place just before and after this time: we know the group of species characteristic of the Neogene that did not survive the Quaternary, and we have information on a group of genera or species that are supposed gradually to have incorporated European ecosystems over a period of about 200,000 years (between 1.9–1.7 Ma) [13]–[15]. The new sites located in the framework of the Fonelas Project provide answers to some of these questions and open new perspectives concerning chronology and place of origin for characteristic taxa of the large mammal dispersal events traditionally considered for Europe.

## Results

### Fonelas Project: Fonelas P-1 (FP-1) site

#### Context

**Figure 1:**
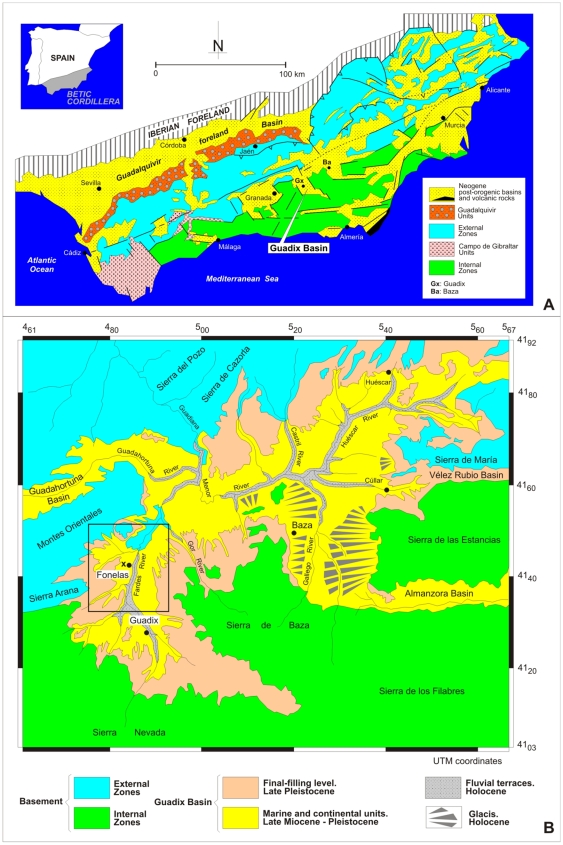
Geological maps of the Betic Cordillera in southern Spain. A. Location of the Guadix Basin in the geologic units of the Betic Cordillera. B. Simplified geological map of the basin, X marks the position of the FP-1 site, in the west slopes of the River Fardes valley (the box delimits the fieldwork area of the Fonelas Project 2001–2007; see Figure S1) (modified after 27).

**Figure 2:**
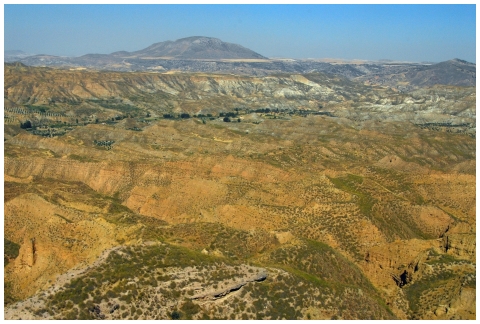
View of the Guadix Basin, to the West (Mencal Hill). Brown rocks belongs to the Internal Transverse System (sterile in mammal fossils); grey and white rocks belongs to the Axial System (units V and VI), fluvial and lacustrine rocks with Pliocene-Pleistocene mammals record.

Together with the north-African Rif, the Betic Cordillera constitutes the western end of the circum-Mediterranean Alpine chains. The Guadix Basin (GB; Figure 1) is situated at the contact between the Internal and External Zones, in the central sector of the Betic Cordillera, where it covers 4,600 km^2^. The basin fill is divided into six genetic units [20], whose bounding discontinuities relate to tectonic events and eustatic change [21]. Our investigation is restricted to the endorheic continental sediments of Units V and VI (Upper Pliocene-Lower Pleistocene). Three depositional systems can be differentiated (Figure 2) according to their palaeogeographic position and the source area of the terrigenous sediments [22], [23]. Along the longitudinal axis of the basin an Axial fluvial system (AS) is composed of gravels, sands and lutites deposited in a river valley, along with palustrine carbonates and evaporites from ephemeral lakes (Figure 1). At the southern margin of the GB the Internal Transverse System is represented by extensive, slightly sloped alluvial fans fed from the hills of the Internal Zones. At the northern edge, the External Transverse System is represented by alluvial fans of small radius and severe slope fed from the hills of the External Zones. The fans of the transversal alluvial systems prograde onto the fluvial deposits of the AS [24], hindering drainage and causing ephemeral lakes to form along the axial valley.

The FP-1 large mammals site, discovered in 2000 and excavated in 2001, 2002, 2004 and 2007 [25], [26], is found in sediments of the axial fluvial system at the top of Unit V. Detailed sedimentological analyses have revealed that the bearing sediments of site FP-1 represent the distal parts of the fluvial drainage system [27], near its connection to a shallow lake. More specifically, the site is located in a sedimentary cycle typical of a meandering fluvial system, and the main accumulation of large mammal fossils is found in a new facies (Facies E, Trench B) genetically independent of purely fluvial processes. This facies is interpreted as the result of bioturbation of a soft substratum by the continuous use of the space by large mammals (carnivorous scavengers, specifically hyaenids -*Pachycrocuta brevirostris*-; the fossil bearing layer has an average thickness of 20 cm).

The detailed palaeogeographic context inferred for the site is that of an abandoned meander. This would have described a slight topographic depression that was periodically flooded, either by rainfall or by slight overflow from the distant active channel, and occupied by large mammals.

**Figure 3:**
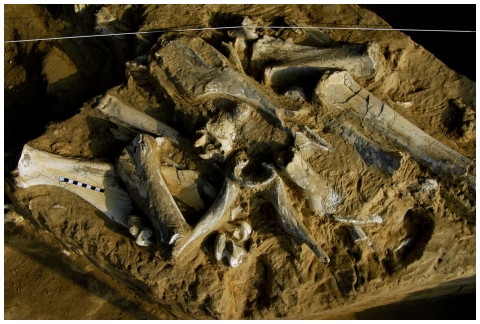
Plan view of part of Fonelas P-1 site (Trench B) with skulls of *Gazellospira* and *Metacervoceros* (both of them gnawed by hyaenids) and long bones of *Equus* and *Mammuthus* (juvenile individuals gnawed by hyaenids).

Facies association E [27] is a ribbon-shaped body 25–40 cm thick and tens of metres wide mainly oriented SW–NE. The base has a very irregular morphology, while the top is planar and horizontal. Seen in cross-section, the boundary surface of this body has vertical walls with scalloped morphology (small saw-toothed cavities) tens of centimetres thick. The most characteristic lithofacies of this body are subangular, very irregularly sized clasts of mud from low facies association C (abandoned channel; [27]) held in a matrix of sand, clay and silt. It has no internal organization (a massive or chaotic structure) and there are some rather thin layers of sand not more than 3 cm thick with ripples similar to the sandy intervals in facies association C. The importance of association E at the Fonelas P-1 site is that it contains the large-mammal fossil concentration (Figure 3) with all the elements and a high diversity of species (this unit has stratigraphic integrity, internal coherence and shows strict short-term contemporaneousness of the fossil assemblage).

#### Magnetostratigraphy of the FP-1 section

The 118 m thick FP-1 section was sampled at aproximatelly 1 m intervals with a total number of 135 magnetostratigraphic sites. Sampled lithologies consisted of fluvial dark grey weakly consolidated sandy silts, alluvial redish-brown silts, palustrine-lacustrine limestones towards the top. The NRM (Natural Remanent Magnetization) shows a wide range of values from 0.03 mA/m to 236 mA/m. Minimum and maximum magnetizations corresponded to limestones and brown-red siltstones respectively, while the relatively high average remanence of 20 mA/m was representative of the grey sandy silts, as it was the predominant sampled lithology. The overall strong magnetization of the the FP-1 sediments likely reflects the high concentration of Fe-oxides in the high-grade metasediements of the Sierra Nevada massif, the source area of the Axial fluvial system.

**Figure 4:**
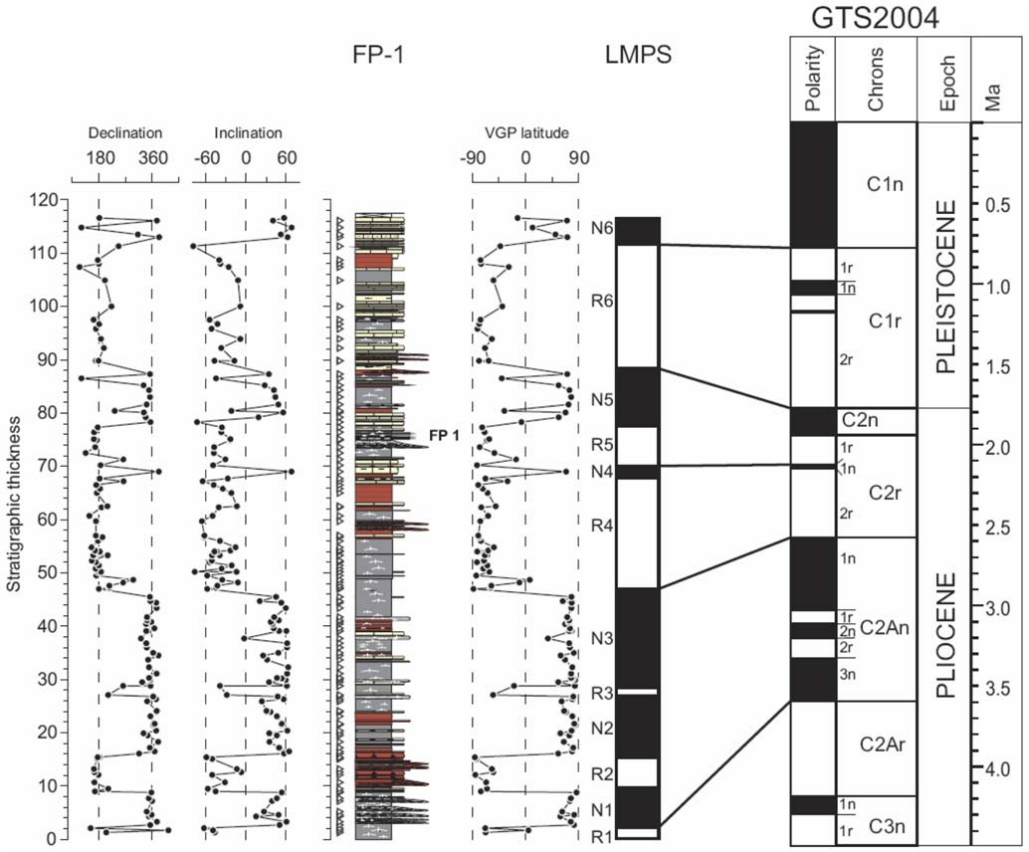
Magnetostratigraphy of the FP-1 section and correlation with the geomagnetic polarity time scale.

The resulting FP-1 section magnetostratigraphy consist of 12 magnetozones, the characteristic pattern of reversals allowing a feasible correlation with the geomagnetic polarity time scale [28], provided the Plio-Pleistocene age of the bearing fossil sites (Figure 4). The 3 thick normal magnetozones N1-N2-N3 in the lower part of the section correlate with the Gauss epoch (C2An) while the dominantly reversed upper part of the section fits well with the Matuyama epoch. Short normal magnetozones N4 and N5 represent Reunion (C2r.1n) and Olduvai (C2n) subchrons respectively. Correlation of the topmost normal magnetozone N6 with Brunhes epoch is preferred because of the existence of archaeological sites of middle Pleistocene age (Solana del Zamborino) associated to the top levels of the basin infill. The obtained magnetostratigraphic correlation yields an age for the FP-1 mammal site of 2.0 Ma, slightly older than the Olduvai subchron.

#### Faunal assemblage at FP-1 (Trench B)

**Figure 5:**
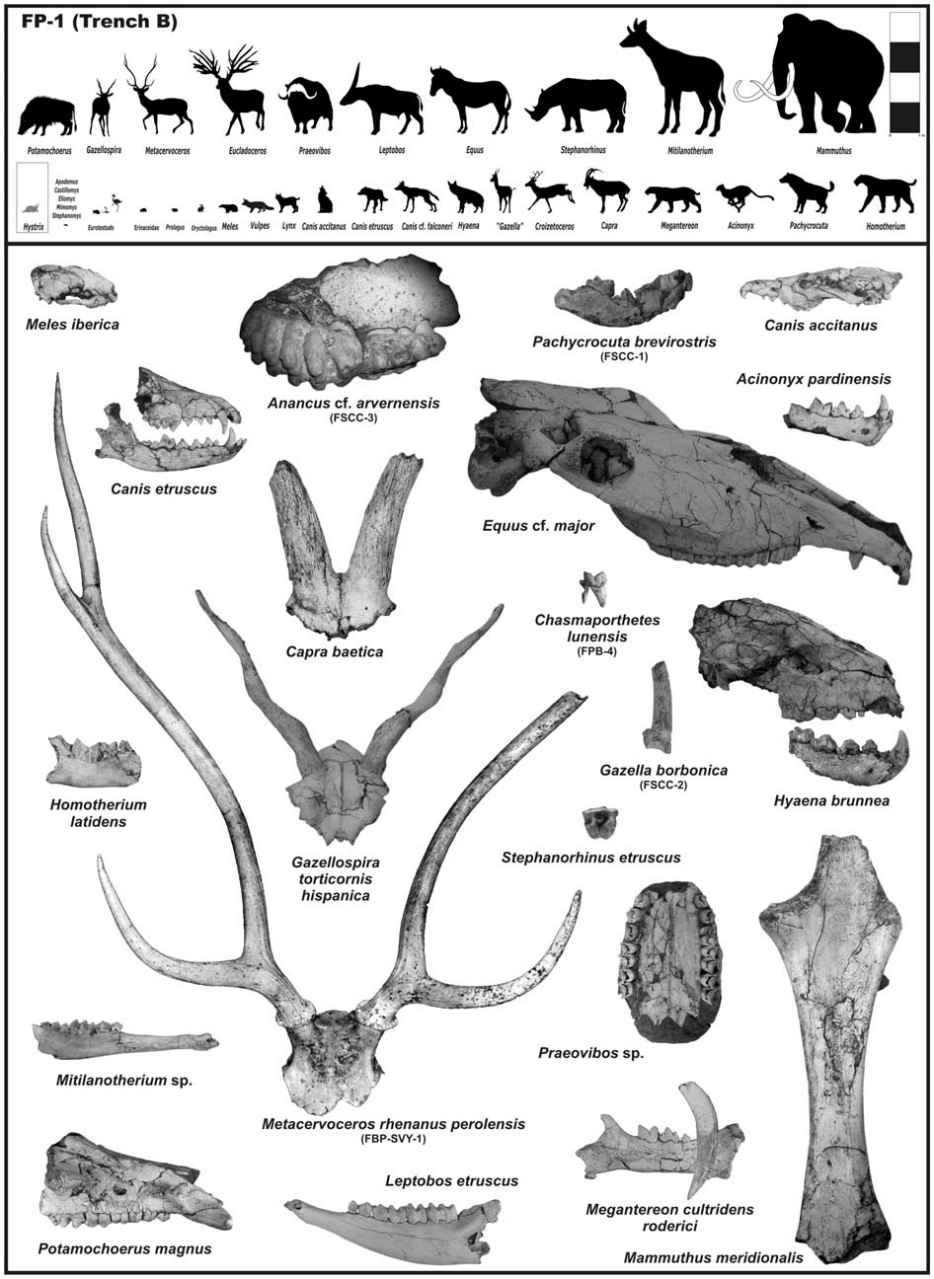
Top: faunal diversity of FP-1 (Trench B). Silhouettes (to scale) of the mammals identified in the deposit (taxonomic discussion in 29, 30, 32, 33). In grey: the taxa (unpublished) identified from indirect taphonomic evidence, as *Hystrix*. Bottom: several fossil specimens (not to scale) of interest from taxonomic, biostratigraphic or palaeobiogeographic points of view, both in FP-1 and other sites located in the work zone since 2001 (see Figs. S1 and S4).

Being only a small part of the studied site (∼800 m^2^), the ca. 40 m^2^ yielded 3000 specimens, 84% of which morphologically and taxonomically identifiable (Figure 5), and many anatomically complete. The abundance and preservation of the Fonelas P-1 large mammal fossils are unique for the Plio-Pleistocene of Iberia.

By the present moment, detailed taxonomic study of the site has identified (Trench B) 38 vertebrates with 32 species of mammals [29], [30] (see supporting information (SI) Table S1): *Meles iberica*, *Canis accitanus*, *Canis etruscus*, *Canis (Xenocyon)* cf. *falconeri*, *Megantereon cultridens roderici*, *Hyaena brunnea*, *Pachycrocuta brevirostris*, *Croizetoceros ramosus fonelensis*, *Capra baetica*, *Gazellospira torticornis hispanica*, *Leptobos etruscus*, *Praeovibos* sp., *Mitilanotherium* sp. and *Potamochoerus magnus*. There is also indirect taphonomic evidence of *Hystrix* (toothmarks) and other biostratinomic agents. Rodent bones in the deposits are scarce, which agrees with the proposed genetic model [27], and were probably incorporated in the record through the faeces of small carnivores. The large mammal assemblage is heterogeneous and novel compared with the associations previously identified for zones MNQ18 and MNQ19 [15], [17], [31].

New species and sub-species were identified [30], [32]: *Meles iberica* (Figure 5; a small badger with an anatomy reminiscent from the Pliocene badger *Meles thorali*); *Megantereon cultridens roderici* (Figure 5; the largest representative of this species, derived from *M. c. cultridens*, with the specific biomechanical characteristics of its jugal dentition); *Croizetoceros ramosus fonelensis* (the smallest and most recent species of the Iberian Villafranchian, an allopatric variety close to *C. r. minor* of Senèze); and *Gazellospira torticornis hispanica* (Figure 5; with the most graceful and recent representatives of the genus in western Europe).

From exceptional skeletal material [29], [30], [33], species of Asian origin new to Iberia but previously known from other countries, or new to science but with possible Asian ancestors, have also been identified: *Canis etruscus* (Figure 5; the only verified citation in Atlantic Europe of the species that defines the “Wolf event”); *Canis accitanus* (Figure 5; the smallest Eurasian jackal, with a mixed canine-vulpine masticating apparatus possibly derived from the first Pliocene populations of *Canis arnensis* [33]); *Capra baetica* (Figure 5; the oldest mountain goat); *Leptobos etruscus* (Figure 5; the only known population of the species in the Iberian Peninsula); *Praeovibos* sp. (Figure 5; whose jugal dentition is more primitive than that of other Lower Pleistocene representatives of the genus); *Mitilanotherium* sp. (Figure 5; the only known population of this Paleotraginae giraffid between Greece and the Atlantic Ocean; fossils of this giraffid are also present in: Romania, Turkey, Georgia and Tajikistan).

Finally, there are species whose evolutionary development comes through the African continent [29], [30]: *Canis (Xenocyon)* cf. *falconeri* (supposed ancestor of the genus *Lycaon*); *Hyaena brunnea* (Figure 5; the south African brown hyena, anatomically distinguishable from *P. perrieri*, never previously identified in the European continent and whose FAD - First Appearance Datum – is provided by the Kenyan site of Kanapoi); *Pachycrocuta brevirostris* (the FAD of which is provided by the Ethiopian site of Hadar, the citation in FP-1 being the oldest for this species in Europe); *Potamochoerus magnus* (Figure 5; this African bush pig is represented by large specimens and this is the first citation of the genus outside Africa).

This large mammal assemblage is unique. It only partially fits the subdivisions of the Villafranchian of France (MNQ system) or Italy (FU system), the reference for the European Plio-Pleistocene paleontological record. It has greater affinity with Zone MNQ18 [15], [31] than with previous or subsequent zones, and can only be described as a mammalian “Lost World” never before hinted at in the fossil record.

### Fonelas Project: Other palaeontological sites in the Fonelas area (2001–2006)

Although FP-1 has been the project's reference site since 2001, our team's aim is to arrive at full understanding of the fossil record in an unexplored portion of the basin measuring 100 km^2^. After four campaigns of systematic palaeontological surveys of the basin's fill (based on stratigraphical, sedimentological and taphonomic criteria), we identified a total of 49 sites with large mammal fossils (Figure S1).

Of these new sites, mainly located in the fluvial units of AS, six are of great biostratigraphic and/or taxonomic interest [26]. From oldest to most recent, they are (Table S1): i) Fonelas SCC-3 (Figure 5; Zone MNQ17); ii) Fonelas SCC-2 (Figure 5; Zone MNQ17); iii) Fonelas PB-4, which for the first time in the GB, yielded remains of one of the hyaenids characteristic of the Upper Pliocene (MNQ17), *Chasmaporthetes lunensis* (Figure 5), together with abundant coprolites (palynologically sterile); iv) Fonelas SCC-1 with 15 taxa, some of them are very significant (Figure 5; bearing in mind that Fonelas SCC-1 shares a wide range of taxa with FP-1, to which from a lithostratigraphic point of view is close, it can be concluded that both sites are of approximately the same age, Zone MNQ18); v) Fonelas BP-SVY-1, with an exceptional neurocranium and well preserved antlers of *Metacervoceros rhenanus perolensis* (Figure 5), a variety characteristic of Zone MNQ19 [16]; and vi) Mencal-9 (M-9) composed of several fluvial and lacustrine layers of the AS with numerous fossils of *Chelonia* gen. indet., *Equus* sp1., *Equus* sp2., Bovidae gen. indet.1, Bovidae gen. indet.2 and *Mammuthus* cf. *meridionalis* (Zones MNQ19 or MNQ20).

The findings made since 2001 within the Fonelas Project as a whole indicate that a set of rock bodies in AS units of the Guadix Basin, with a mean thickness of 6 m and localized between 920–940 m a.s.l., contain valuable information on the mammals communities of the later Pliocene (Zone MNQ17: Fonelas SCC-3, Fonelas SCC2, Fonelas BP-4; Zone MNQ18: FP-1, Fonelas SCC-1), the Plio-Pleistocene boundary (Zone MNQ19: Fonelas BP SVY-1, Mencal-8) and the beginning of the Lower Pleistocene (Mencal-9). These units occupy a minimum surface area of 40 km^2^ and comprise a volume of about 240,000,000 m^3^ of sedimentary rocks with a stratigraphic record of paleoenvironments and a fossil record of large mammals characteristic of terrestrial ecosystems.

## Discussion

### Implications for biostratigraphy and palaeobiogeography

**Figure 6:**
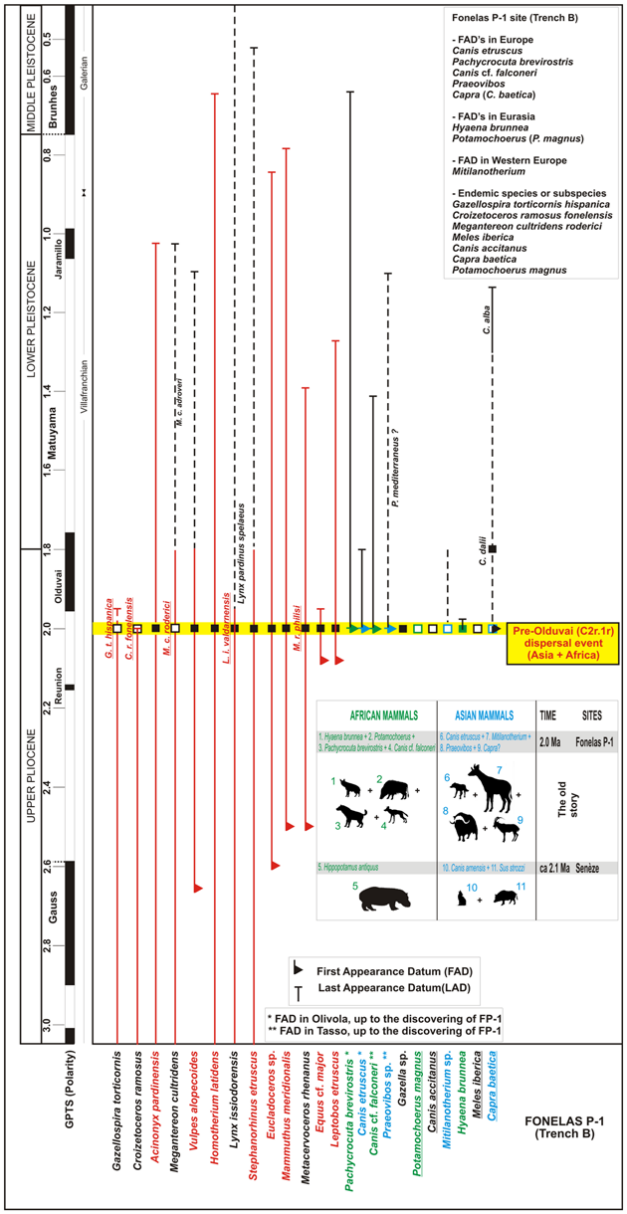
Biostratigraphic summary of the assemblage of large mammals in FP-1 (the position of FP-1, Trench B, is the result of the magnetostratigraphic investigation presented here). The integral analysis of the information obtained of the FP-1 palaeomastologic assemblage permits us: to establish that the singular assemblage of FP-1 is slightly older (2.0 Ma) than the Olivola + Tasso FU; to observe the noticeable increase in the FADs in the Fonelas P-1 (Trench B) assemblage; to verify the existence of zone MNQ18 in chronologies older than Olduvai; and to put forward the hypothesis of one *pre-Olduvai (C2r.1r) dispersal event* between 2.1 and 2.0 Ma that would bring together the Asian and African large mammals (the old story on the configuration of European pre-Pleistocene ecosystems). Future research at FP-1 and the unpublished site of M-9 might permit us to verify the presence of different primates in the GB in so early chronologies.

The genera and species identified in FP-1 (Trench B) and the set of associated sites permit a chronostratigraphic assignment of the site to the end of the Upper Pliocene and, in turn, sheds light on the biochronology and palaeobiogeography of the N–Q transition in Eurasia (Figure 6 and supplementary information in Figure S4). Two major sea-level drops stand out in this time interval, owing to the wide-ranging environmental changes that accompanied them in the continental area [34]: the Aquatraversan and the Aullan erosional phases. In terms of fossil plio-pleistocene dispersals, the traditional interpretation [34], [35], is that:

- The first immigration, nominally defined as “the Elephant-*Equus* dispersal event”, can be placed between 2.6–2.5 Ma and has an Asian origin. The event is characterized by the incorporation in the record of *Eucladoceros*, monodactyl equids (*Equus*), another medium-sized cervid (*Metacervoceros*) and the first elephants of the genus *Mammuthus*. This dispersal would signify the replacement in the continental ecosystems of Europe of the mastodons of the genus *Anancus* by the first elephants (despite the fact that some subsequent deposits show the coexistence of both proboscideans, for example at Saint Vallier) and of the tridactyl equids (*Hipparion*) by monodactyls.
- The second immigration, for which the biostratigraphic setting of the FP-1 site is most relevant, is known as the *“Wolf event”*, which also has an Asian origin. This event primarily concerns the species *Canis etruscus* [modern canid, whose first certain data come from the Olivola Faunal Unit (Olivola FU), even though the genus is already present in Senèze] and by the incorporation in the European palaeomastological record of *Sus strozzi* (the first data also from Senèze). However, other immigrants also colonized European ecosystems, such as the short-faced hyena *Pachycrocuta brevirostris*, very probably African in origin [13]. Defined at the Olivola FU (Upper Valdarno, Italy), this dispersal event has been placed at 1.9 Ma and related with the so-called *“Homo event”* [13], [36], [37], as recorded at the Caucasian site of Dmanisi.
- The last dispersal event defined in relation with the Plio-Pleistocene boundary is that characterized by the Tasso Faunal Unit (Tasso FU, Upper Valdarno, Italy), close to 1.7 Ma [38]. Again, Asian species would be incorporated into European ecosystems, such as the great ovibovine *Praeovibos*, ancestor of current muskox, and the small jackal, *Canis arnensis*, together with until then exclusively African species, such as the hippopotamus (*Hippopotamus antiquus*) and the wild dog [*Canis (Xenocyon) falconeri*].

Based on the FADs and LADs (Last Appearance Data) of the different taxa identified in the site, this historical interpretation of the European fossil record would assign FP-1 to the period between 1.9 and 1.7 Ma [25], [27], [32], [33]. This is because the Spanish assemblage should not be older than the Olivola FU in Italy (1.9 Ma), where we find the FADs of several large mammals known to be from Asia. However, the magnetostratigraphic data presented above date FP-1 to about 2.0 Ma.

In FP-1 (Trench B), species supposedly specific to the *Wolf event* (FADs of 1.9 Ma) coexist with species characteristic of the most recent of the dispersal events proposed for this chronological period (Tasso FU; FADs of 1.7 Ma). FP-1 thus confirms the coexistence (and, therefore, the prior immigration in the late Upper Pliocene) of species assigned to the *Wolf event* (*Canis etruscus*+*Pachycrocuta brevirostris*) with representatives of genera or species of presumed later inmigration (*Praeovibos* sp., *Canis accitanus* and *Canis* cf. *falconeri*) (Figure 6). Since the putative protagonists of the Tasso FU are in fact associated with the *Wolf event* (Olivola+Tasso FU's; Figure S4) we contend that there were no dispersal events associated with that FU. Moreover, the age of the *Wolf event* must be that indicated by the magnetostratigraphic dating of FP-1 (Trench B), or slightly older.

The new information provided by the Fonelas sites, complemented by Senèze and Dmanisi, points to an early incorporation of Asian and African taxa into European ecosystems in the framework of a dispersal event for which we propose the formal designation of the *pre-Olduvai (C2r.1r) dispersal event* (ca. 2.1–2.0 Ma). This event would embrace (Figure 6, Figure S4): i) the historical *Wolf event*, whose basic association of large mammals includes *Canis arnensis* (sin. *C. senezensis*)−*Canis accitanus*+*Canis etruscus*+*Praeovibos* sp.+*Sus strozzi*; and ii) the *Homo event*, contemporaneous with the above mentioned event and introducing to European ecosystems *Pachycrocuta brevirostris*+*Hippopotamus amphibius* (the identification of *Hippopotamus* in Senèze makes sense in this scenario [39])+*Canis falconeri*+*Homo* (*H.* aff. *erectus*) ¿+? *Theropithecus oswaldi*. In this sense, FP-1 adds new taxa from both Asia and Africa to this dispersal event: from Asia, *Mitilanotherium* and possibly *Capra* (although FP-1 holds the FAD of the genus at the present time), and, from Africa, *Hyaena brunnea* and *Potamochoerus*.

The palaeobiocoenosis of FP-1 (Trench B) thus suggests that dispersal events hitherto considered separately [those initially defined in the Olivola (*Wolf event*) and Tasso FUs] are in fact one, and one that belongs in the conventional Zone MNQ18 which should be redefined accordingly. Zones MNQ17, MNQ18, MNQ19 and, perhaps, Zone MNQ20 have been identified within the same general succession of the GB. Future excavations and progress in the investigation will clarify whether the presence/absence of fossil remains of *Hippopotamus*, *Theropithecus* and other primates in these deposits is due to taphonomic biases, to variables derived from past ecological exclusions, or to the real absence of their populations from the south of the Iberian Peninsula at this time.

The geographical situation and chronology of Fonelas P-1 opens up palaeobiogeographic uncertainties. The earliest records of species of supposed Asian origin are now in the southwest of the European continent, and no comparable palaeontological evidence is known west of the Caucasus (Georgia). The palaeobiogeographic singularity of the Iberian Peninsula during the late Upper Pliocene would be confirmed by the presence in GB of species of African lineage, and by the presence in North African sites of large mammals characteristic of the European Upper Pliocene, in both Atlantic (Ahl Al Oughlam, Upper Pliocene [40]; *Ursus* cf. *etruscus*, a new species of the genus *Nyctereutes* and a new variety of *P. perrieri*) and Mediterranean regions (Ain el Hanech, Plio-Pleistocene [41]; *Mammuthus meridionalis*).

These patterns of large mammal exchanges, potentially across the Strait of Gibraltar, open up the possibility that hominins dispersed into Europe via the Maghreb (in Atlantic regions) and Iberia, perhaps explaining why it is only in Iberia that secure evidence of a human occupation of Europe prior to 1 Ma is known (archaeological sites in GB, Atapuerca/Sima del Elefante). This issue of potential spread of large mammals across the Gibraltar Strait has been often addressed simplistically [42]. In the specific case of the period between 3.0 and 2.0 Ma [43], we certainly need to cohere the existing palaeobiological information from the Iberian Peninsula and North Africa with a contending model of the geologic history of the Strait [44], [45], especially with reference to underwater geomorphology, which is largely unknown for the Upper Pliocene.

An alternative interpretation is that the African taxa in FP-1 represent late survivals of the older Pliocene or younger Miocene faunal communities whose distribution range encompassed both Africa and the Greater Mediterranean. This interpretation is, however, inconsistent with the fact that these mammals remain undocumented in the older Pliocene sites of Iberia. Given these considerations, the only real alternative to the Upper Pliocene faunal exchanges across the Straits is that of a dispersal of the African taxa along the coasts of the northern Mediterranean. Acceptance of this view, however, requires an explanation for their absence at Dmanisi and at the Italian and French sites of the period. The Iberian Peninsula will play an essential role in understanding this dispersal event between Asia and Europe and between Africa and Europe in these chronologies, since our research has increased the number of possible routes for the longitudinal and latitudinal dispersal of the different taxa involved.

## Materials and Methods

### Magnetostratigraphy

Samples were collected using an electric drill and cores were oriented in the field using a magnetic compass. Standard paleomagnetic samples were processed in the Paleomagnetic Laboratory at the Institute of Earth Sciences Jaume Almera (CSIC-University of Barcelona). The NRM was mesured in a three-axis superconducting magnetometer (2G SRM 750).

Stepwise thermal demagnetization was applied to all samples in order to determine the different components of the NRM. A Schonstedt TSD1 non inductive two-chamber furnace was utilized to demagnetize the samples at 50° to 20° thermal steps up to complete removal of the remanence (Figure S2). Magnetic susceptibility (MS) was measured after each temperature step in order to monitor changes in the rock magnetic mineralogy. The observed MS records indicate that no significant mineralogical changes occur upon heating to 400°C. Sudden increase of MS at temperatures above 400°C and peaking at 500°C points to the formation of significant amounts of magnetite during thermal demagnetization. Fortunately, the low residual field (<10 nT) kept in the interior of the demagnetizer allowed that the magnetic remanence resulted unaffected by these mineralogical changes.

Stepwise thermal demagnetization results were analyzed by visual inspection of the Zijderveld diagrams (Figure S2). Most diagrams illustrate the presence in all the samples of a northward directed component with maximum unblocking temperatures of 250°C which may represent from 50% to 95% of the NRM. This soft component is subparallel to the present day field and is thus interpreted as a recent overprint acquired during the Brunhes period. At temperatures above 250°C samples show a single linear component which gradually decays up to maximum unblocking temperatures of about 600°C or even higher. We interpret this characteristic component as carried by a mixture of both magnetite and hematite as part of the detrital fraction of the studied sediments.

The orientation the characteristic magnetization yielded a dual-polarity distribution with antipodal normal and reverse polarity means (Figure S3). The angle between the mean of the normal and reverse polarity sets is 6.8°, which is less than the critical angle 8.4° determined following the reversal test of [46]. These results represent a positive reversal which indicates that laboratory treatment was successful in isolating the characteristic component from the recent overprint. Both normal and reverse mean directions yielded inclinations significantly shallower than expected for the geographic latitude of the FP-1 section (Figure S3). We interpret such byass as a post-depositional flattening of an early acquired detrital remanence.

Virtual Geomagnetic Pole (VGP) latitude was calculated for each site in order to establish a Local Magnetic Polarity Stratigraphy (LMPS) of the FP-1 section, positive VGP latitudes corresponding to normal polarity magnetization and negative VGP latitudes corresponding to reverse polarity magnetization.

## Supporting Information

Figure S1 Detailed map of the work area (see Figure 1) of Fonelas Project for 2001–2007 (built up areas in black: Fonelas, Benalúa, Belerda and Pedro Martínez). Position of both sites known before the project was undertaken (Huélago and La Solana del Zamborino), the position of the new large mammal sites located in the Fonelas Project and the location of the stratigraphic successions studied in the analyzed sector. The channelling of the Fardes River has produced a landscape of badlands, between 1,000 m and 700 m, and opened up a spectacular fossil-bearing outcrop of the Plio-Pleistocene.
(4.05 MB TIF)

Figure S2 Demagnetization vector endpoint diagrams of representative samples of the FP-1 section. Inset plots represent remanent magnetization (black dots) and susceptibility (white dots) measured at room temperature after each demagnetization step.
(1.42 MB TIF)

Figure S3 Equal area stereonet projection of paleomagnetic directions of the FP-1 section and fisher statistics of the mean normal and reverse directions. Red stars represent the expected Geocentric Axial Dipole directions at the site.
(0.39 MB TIF)

Figure S4 Summary of the biostratigraphic information of FP-1 with data of other Spanish and European sites. The Spanish, European and Caucasian sites are situated in the most parsimonious positions as a function of the research carried out into each. The integral analysis of the information permits us also: to integrate into one the dispersal events traditionally assigned to the Olivola and Tasso FUs; to establish that the singular assemblage of FP-1 is slightly older (−2.0 Ma) than the Olivola + Tasso FU (according to our proposal in the figure); to observe the noticeable increase in the FADs between Senéze and Fonelas P-1 (Trench B), that is, 2.1–2.0 Ma old; and to reinforce the hypothesis of one pre-Olduvai (C2r.1r) dispersal event between 2.1 and 2.0 Ma, characterized by the “Wolf” dispersal event (1 in the figure) plus those associated with the “Homo” dispersal event (2 in the figure), to which other new protagonists can be added thanks to the FP-1 record. The remaining deposits complement the hypothesis proposed in that records like those of Pirro Nord (−1.3±0.1 Ma), Barranco León-5+Fuente Nueva-3 (−1.2±0.1 Ma) and Sima del Elefante (−1.1±0.2 Ma) represent assemblages resulting from the pre-Olduvai (C2r.1r) dispersal event.
(8.40 MB TIF)

Table S1 Vertebrates identified at FP-1 and other new sites at the GB (from oldest, on the left side, to youngest, on the right). *, marks the sites with hyaenid activity.
(1.00 MB DOC)
