# Supplementary figures and images for: A Mammalian Lost World in Southwest Europe during the Late Pliocene
Source: PLoS One. 2009 Sep 23;4(9):e7127. doi: 10.1371/journal.pone.0007127 (PMC2745751; doi:10.1371/journal.pone.0007127)

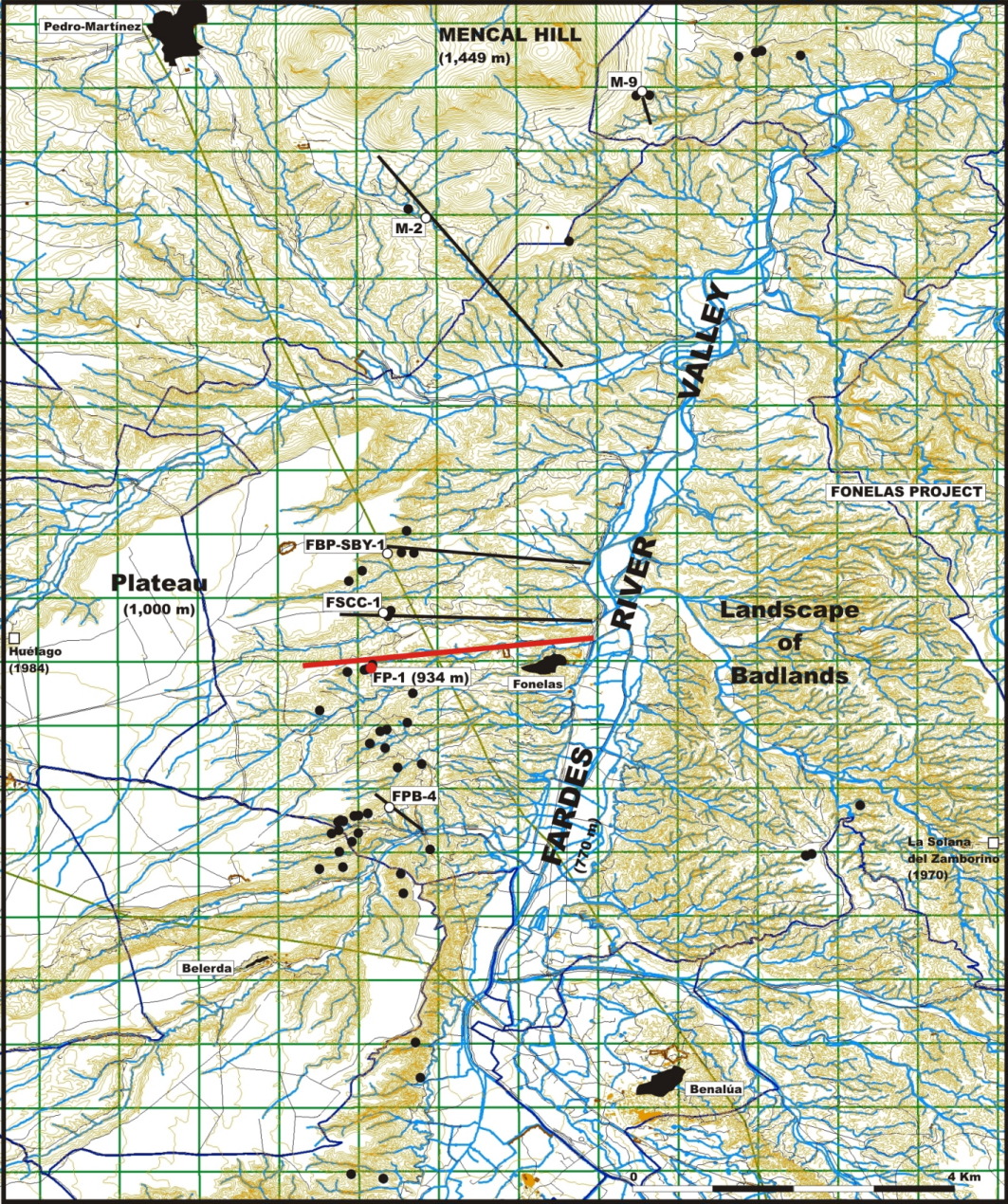

Supplement: Figure S1 — Detailed map of the work area (see Figure 1) of Fonelas Project for 2001–2007 (built up areas in black: Fonelas, Benalúa, Belerda and Pedro Martínez). Position of both sites known before the project was undertaken (Huélago and La Solana del Zamborino), the position of the new large mammal sites located in the Fonelas Project and the location of the stratigraphic successions studied in the analyzed sector. The channelling of the Fardes River has produced a landscape of badlands, between 1,000 m and 700 m, and opened up a spectacular fossil-bearing outcrop of the Plio-Pleistocene. (4.05 MB TIF) [file pone.0007127.s001.tif]

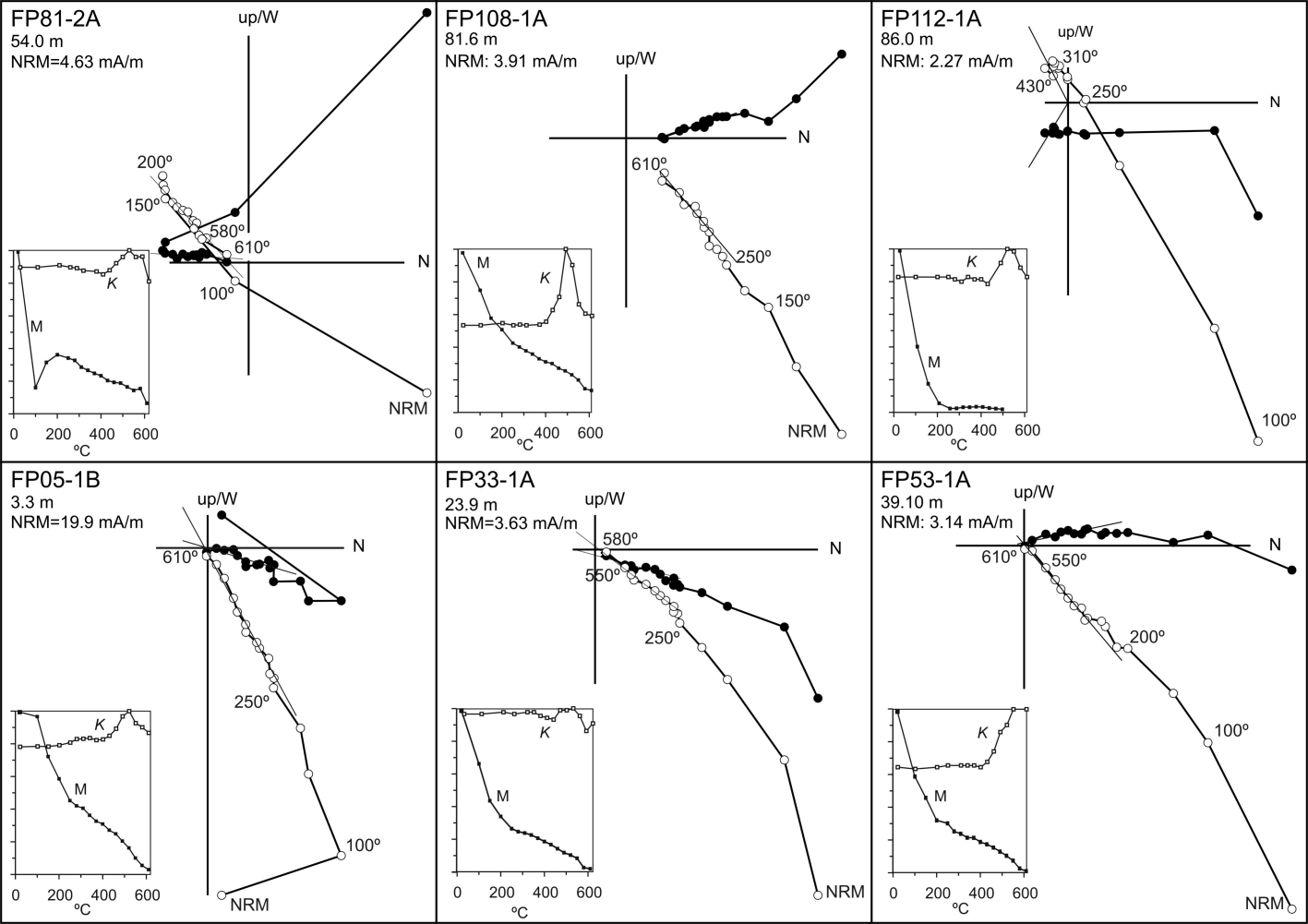

Supplement: Figure S2 — Demagnetization vector endpoint diagrams of representative samples of the FP-1 section. Inset plots represent remanent magnetization (black dots) and susceptibility (white dots) measured at room temperature after each demagnetization step. (1.42 MB TIF) [file pone.0007127.s002.tif]

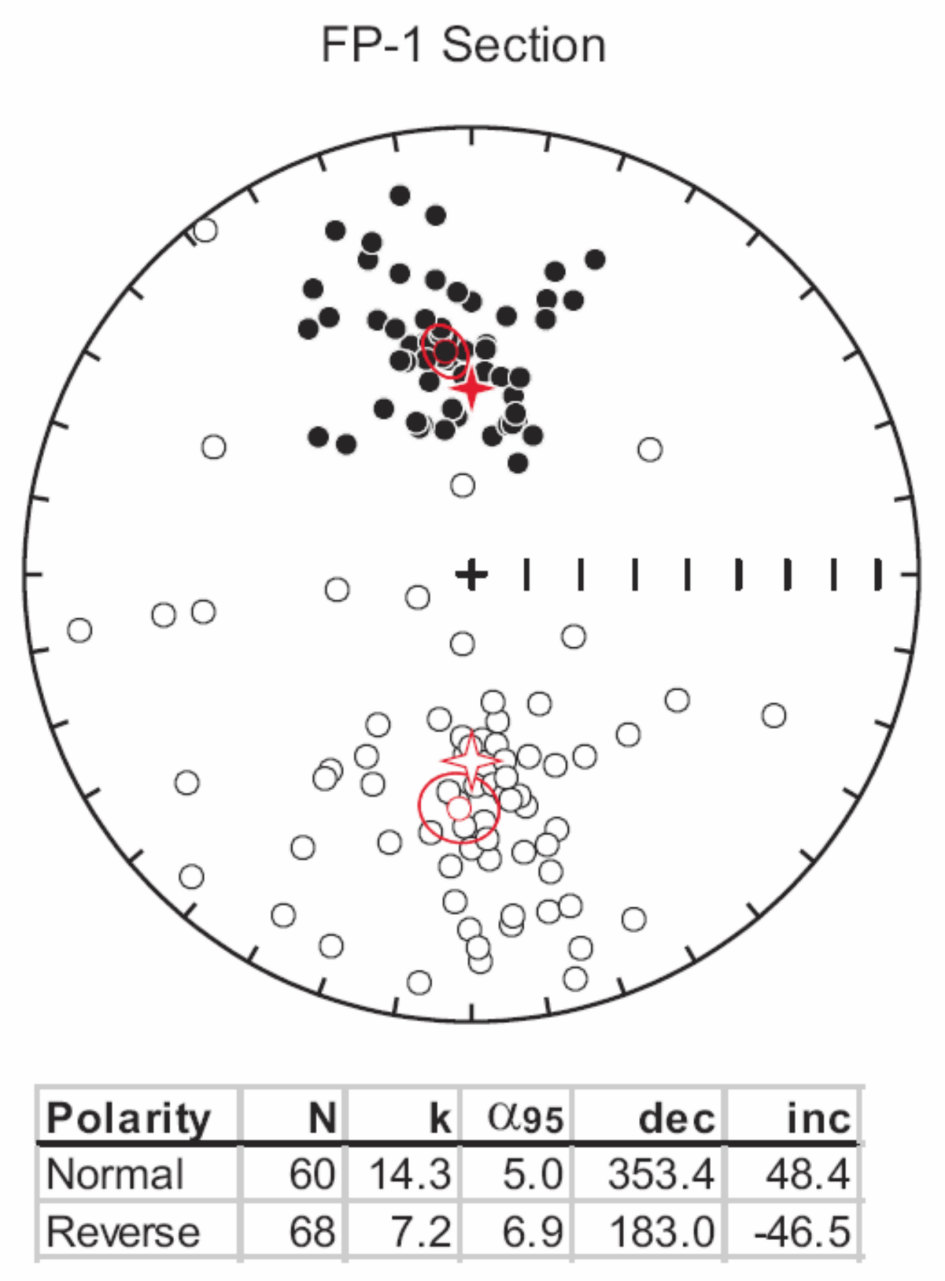

Supplement: Figure S3 — Equal area stereonet projection of paleomagnetic directions of the FP-1 section and fisher statistics of the mean normal and reverse directions. Red stars represent the expected Geocentric Axial Dipole directions at the site. (0.39 MB TIF) [file pone.0007127.s003.tif]

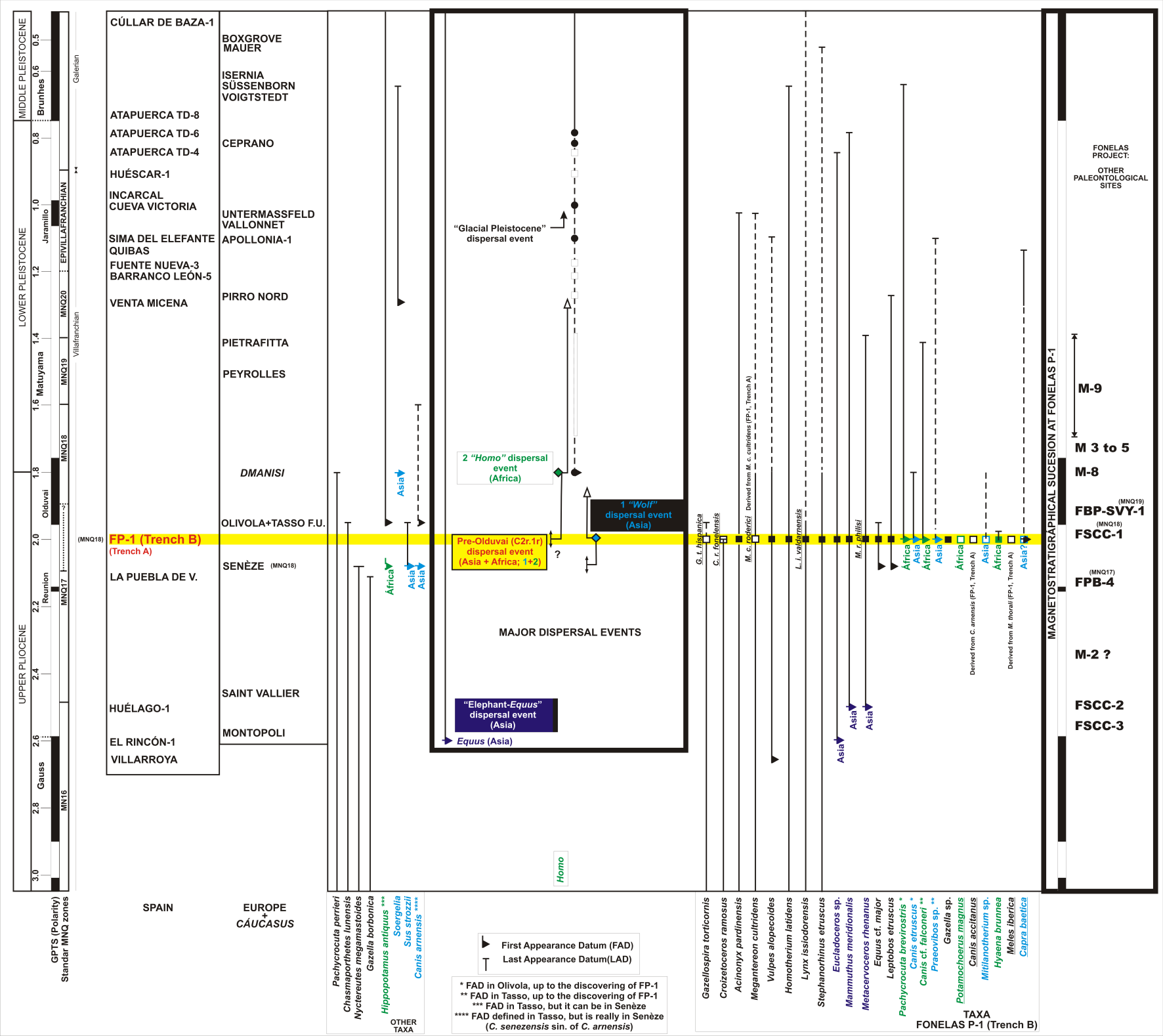

Supplement: Figure S4 — Summary of the biostratigraphic information of FP-1 with data of other Spanish and European sites. The Spanish, European and Caucasian sites are situated in the most parsimonious positions as a function of the research carried out into each. The integral analysis of the information permits us also: to integrate into one the dispersal events traditionally assigned to the Olivola and Tasso FUs; to establish that the singular assemblage of FP-1 is slightly older (−2.0 Ma) than the Olivola + Tasso FU (according to our proposal in the figure); to observe the noticeable increase in the FADs between Senéze and Fonelas P-1 (Trench B), that is, 2.1–2.0 Ma old; and to reinforce the hypothesis of one pre-Olduvai (C2r.1r) dispersal event between 2.1 and 2.0 Ma, characterized by the “Wolf” dispersal event (1 in the figure) plus those associated with the “Homo” dispersal event (2 in the figure), to which other new protagonists can be added thanks to the FP-1 record. The remaining deposits complement the hypothesis proposed in that records like those of Pirro Nord (−1.3±0.1 Ma), Barranco León-5+Fuente Nueva-3 (−1.2±0.1 Ma) and Sima del Elefante (−1.1±0.2 Ma) represent assemblages resulting from the pre-Olduvai (C2r.1r) dispersal event. (8.40 MB TIF) [file pone.0007127.s004.tif]
