# Supplementary material for: A Mammalian Lost World in Southwest Europe during the Late Pliocene
Source: PLoS One. 2009 Sep 23;4(9):e7127. doi: 10.1371/journal.pone.0007127 (PMC2745751; doi:10.1371/journal.pone.0007127)
Supplement: Table S1 — Vertebrates identified at FP-1 and other new sites at the GB (from oldest, on the left side, to youngest, on the right). *, marks the sites with hyaenid activity. (1.00 MB DOC) [file pone.0007127.s005.doc]

| **Taxa** | **Fonelas SCC-3** | **Fonelas SCC-2** | **Fonelas PB-4*** | **FP-1* (Trench B)** | **Fonelas SCC-1*** | **Fonelas BP-SVY-1** | **Mencal-9** |
| --- | --- | --- | --- | --- | --- | --- | --- |
| Lacertidae gen. indet. |  |  |  |  |  |  |  |
| Anguidae gen. indet. |  |  |  |  |  |  |  |
| *Rhinechis scalaris* |  |  |  |  |  |  |  |
| Viperidae gen. indet. |  |  |  |  |  |  |  |
| *Eurotestudo* sp. |  |  |  |  |  |  | ? |
| Aves gen. indet. |  |  |  |  |  |  |  |
| *Mimomys* sp. |  |  |  |  |  |  |  |
| *Castillomys* sp. gr. *C. crusafonti*-*C.* *rivas* |  |  |  |  |  |  |  |
| *Apodemus* cf. *atavus* |  |  |  |  |  |  |  |
| *Stephanomys* sp. |  |  |  |  |  |  |  |
| *Eliomys* sp. |  |  |  |  |  |  |  |
| *Prolagus* cf. *calpensis* |  |  |  |  |  |  |  |
| *Oryctolagus* sp. |  |  |  |  |  |  |  |
| Erinaceidae gen. indet. |  |  |  |  |  |  |  |
| ***Meles iberica*** |  |  |  |  |  |  |  |
| *Vulpes alopecoides* |  |  |  |  |  |  |  |
| ***Canis accitanus*** |  |  | indet |  |  |  |  |
| ***Canis* *etruscus*** |  |  |  |  |  |  |  |
| ***Canis* *(Xenocyon)* cf. *falconeri*** |  |  |  |  |  |  |  |
| *Lynx issiodorensis valdarnensis* |  |  |  |  |  |  |  |
| *Acinonyx pardinensis* |  |  |  |  |  |  |  |
| ***Megantereon cultridens roderici*** |  |  |  |  | ssp |  |  |
| *Homotherium* *latidens* |  |  |  |  |  |  |  |
| ***Hyaena brunnea*** |  |  |  |  |  |  |  |
| ***Pachycrocuta brevirostris*** |  |  |  |  |  |  |  |
| *Chasmaporthetes lunensis* |  |  |  |  |  |  |  |
| ***Croizetoceros* *ramosus fonelensis*** |  |  |  |  |  |  |  |
| *Metacervoceros rhenanus philisi* |  |  |  |  |  |  |  |
| *Metacervoceros rhenanus perolensis* |  |  |  |  |  |  |  |
| *Eucladoceros* sp. |  |  |  |  |  |  |  |
| ***Capra baetica*** |  |  |  |  |  |  | ? |
| *Gazella* sp. |  |  |  |  |  |  |  |
| *Gazella borbonica* |  |  |  |  |  |  |  |
| ***Gazellospira* *torticornis hispanica*** |  |  | sp |  | ssp |  |  |
| ***Leptobos* *etruscus*** |  |  | sp |  |  |  | ? |
| ***Praeovibos* sp.** |  |  |  |  |  |  |  |
| ***Mitilanotherium* sp.** |  |  |  |  |  |  |  |
| ***Potamochoerus magnus*** |  |  |  |  |  |  |  |
| *Equus* cf. *major* |  |  |  |  |  |  | ? |
| *Equus* aff. *altidens* |  |  |  |  |  |  | ? |
| *Stephanorhinus* *etruscus* |  |  |  |  |  |  |  |
| *Mammuthus meridionalis* |  |  |  |  |  |  |  |
| *Anancus* cf. *arvernensis* |  |  |  |  |  |  |  |
